# Bio-Nanovesicle-Based Approaches for Hair and Skin Regeneration: An Updated Concise Review

**DOI:** 10.3390/cells15070617

**Published:** 2026-03-30

**Authors:** Ramya Lakshmi Rajendran, Danyal Reyaz, Atharva Anand Mahajan, Chae Moon Hong, Kandasamy Nagarajan ArulJothi, Byeong-Cheol Ahn, Prakash Gangadaran

**Affiliations:** 1 Department of Nuclear Medicine, School of Medicine, Kyungpook National University, Daegu 41944, Republic of Korea; ramyag@knu.ac.kr (R.L.R.); cmhong@knu.ac.kr (C.M.H.); 2 Cardiovascular Research Institute, Kyungpook National University, Daegu 41944, Republic of Korea; 3 BK21 FOUR KNU Convergence Educational Program of Biomedical Sciences for Creative Future Talents, Department of Biomedical Sciences, School of Medicine, Kyungpook National University, Daegu 41944, Republic of Korea; 4 Department of Genetic Engineering, College of Engineering and Technology, SRM Institute of Science and Technology, Potheri, Kattankulathur, Chengalpattu 603203, Tamilnadu, India; dr6483@srmist.edu.in; 5 Advanced Centre for Treatment, Research and Education in Cancer, Navi Mumbai 410210, Maharashtra, India; atharva.mahajan@actrec.gov.in; 6 Department of Nuclear Medicine, Kyungpook National University Hospital, Daegu 41944, Republic of Korea

**Keywords:** bio-nanovesicles, extracellular vesicles, exosomes, artificial nanovesicles, hair regeneration, Wnt/β-catenin signaling, dermal papilla cells, skin regeneration

## Abstract

Skin and hair follicles regenerate through coordinated stem cell niches and cyclic signaling associated with transitions among anagen, catagen, and telogen phases. In alopecia and chronic skin diseases, follicular miniaturization, immune dysregulation, persistent inflammation, impaired vascularization, and a compromised stratum corneum barrier limit the effectiveness of conventional topical and systemic therapies. Bio-nanovesicles (BNVs), including natural extracellular vesicles such as exosomes and microvesicles, as well as engineered artificial or hybrid nanovesicles, offer a targeted, cell-free delivery platform for miRNAs, proteins, and growth factors. By modulating key pathways—Wnt/β-catenin, PI3K/AKT, MAPK/ERK, and TGF-β/BMP—BNVs have the potential to restore regenerative crosstalk, enhance angiogenesis, and help initiate hair and skin repair.

## 1. Introduction

### 1.1. Overview of the Regenerative Biology of Hair Follicles and Skin

Skin and hair follicle regeneration centers on coordinated interactions between specialized stem cell niches and cyclic molecular signaling. In the skin, regeneration is maintained by basal keratinocytes and dermal fibroblasts that regulate wound healing and tissue homeostasis, while the hair follicles act as self-renewing “micro-organs” undergoing lifelong cycles of growth (anagen), regression (catagen), and rest (telogen). This cyclic regeneration is regulated by multipotent stem cells in the follicular bulge. They are activated by inductive signals from the dermal papilla (DP)—a cluster of mesenchymal cells at the follicle base.

Key pathways, including Wnt/β-catenin, Shh, and bone morphogenetic protein (BMP), act as biological “switches” that determine cell fate and phase transitions [1,2]. Bio-nanovesicle (BNV) therapies leverage this machinery by delivering targeted molecular cargo—such as microRNAs (miRNAs) and growth factors—to specific niches, potentially stimulating regeneration in alopecia or chronic wounds [3].

### 1.2. Challenges in Treating Alopecia and Chronic Skin Diseases

Treating alopecia and chronic skin diseases is challenging due to the complex, multifactorial nature of the skin microenvironment. In alopecia, particularly androgenetic alopecia (AGA) and alopecia areata, key barriers include follicular miniaturization and loss of immune privilege, which can lock the follicles in a prolonged resting state or target them for destruction [4]. Standard pharmacological treatments often have poor bioavailability because the stratum corneum acts as a physical barrier that limits deep penetration of active molecules to the follicular bulge or DP [5].

Furthermore, chronic skin diseases such as diabetic ulcers or psoriasis involve a stalled healing process associated with excessive inflammation, impaired vascularization, and extracellular matrix (ECM) dysfunction. Conventional therapies often fail to address the cellular crosstalk required to resolve inflammation, creating a clinical gap in which topical treatments are insufficient and systemic treatments carry significant side effects. This highlights the need for BNV-based systems that bypass the skin barrier and deliver precise, localized instructions to reset tissue regeneration [3,6].

### 1.3. Introduction to Bio-Nanovesicles: Extracellular Vesicles, Exosomes, and Artificial Nanovesicles

BNVs represent an emerging frontier in drug delivery and regenerative medicine, functioning as biological “envelopes” that transport therapeutic cargo with high precision [7]. These vesicles are broadly categorized into two groups: naturally secreted and engineered.

#### 1.3.1. Extracellular Vesicles

Extracellular vesicles (EVs) are the broad umbrella term for all lipid bilayer-delimited particles naturally released by cells. They are essential for intercellular communication, acting as messengers that carry proteins, lipids, and nucleic acids, such as messenger RNA and miRNA, from donor to recipient cells [8,9].

#### 1.3.2. Exosomes

Exosomes are a specific subpopulation of EVs, typically ranging 30–150 nm in diameter. Unlike vesicles that bud directly from the plasma membrane, they originate from the endosomal pathway (multivesicular bodies, MVBs). Their small size and distinct surface markers (such as CD63 and CD81) enable deep tissue penetration and passage across biological barriers that larger particles cannot cross [10,11].

#### 1.3.3. Artificial Nanovesicles

To overcome limitations of natural production, such as low yield and complex purification, researchers have developed bio-inspired or artificial nanovesicles. These include liposomes (synthetic lipid bilayers) and cell-derived nanovesicles (CDNs) created via mechanical cell fragmentation [12]. Artificial vesicles can be loaded with drugs or engineered with surface ligands to target hair follicle stem cells (HFSCs) or inflamed skin tissue [13,14].

To maintain scientific precision, this review distinguishes three key terms: EVs, naturally occurring lipid bilayer particles released by cells for communication; artificial nanovesicles, synthetic or mechanically engineered mimetics such as liposomes or CDNs; and BNVs, an overarching functional term for the combined therapeutic platform of natural and engineered vesicles used in dermatology.

### 1.4. Rationale for Bio-Nanovesicle-Based Therapies

The rationale for BNV-based therapies in dermatology lies in their ability to serve as superior cell-free alternatives to traditional treatments and live cell injections. BNVs, particularly exosomes, provide high biocompatibility and low immunogenicity by utilizing natural membranes, enabling them to bypass the stratum corneum barrier that typically limits larger or more polar molecules. Their nanoscale size and capacity for surface engineering enable precise targeting of deep regenerative niches, such as the hair follicle bulge and DP. Furthermore, the BNV lipid bilayer protects delicate cargo—including miRNAs and growth factors—from enzymatic degradation, preserving functional integrity upon delivery [5,15]. By capturing the therapeutic effects of stem cells without the risks of immune rejection, tumorigenicity, or poor cell survival, BNVs provide a stable, standardized, and potent platform for restoring regenerative cycles in skin and hair appendages [16,17,18].

While early reviews establish the role of exosomes in dermatology, this updated, concise review reflects the recent surge in human clinical data (2025–2026) and the shift toward hybrid engineered vesicles. It also integrates emerging computational tools and bioprinting in personalized BNV delivery, providing a contemporary roadmap for clinical translation.

### 1.5. Literature Search Strategy

To ensure a comprehensive and up-to-date review, a systematic literature search was conducted across major databases, including PubMed, Scopus, Web of Science, and Google Scholar. The search was limited to articles published between January 2014 and January 2026 to capture key advances in nanovesicle engineering and clinical translation. Search keywords: Combinations of the following terms were used: “bio-nanovesicles,” “extracellular vesicles,” “exosomes,” “engineered nanovesicles,” “hair follicle regeneration,” “dermal papilla cells,” “skin regeneration,” “alopecia,” and “chronic wound healing.” Final scope: After full-text review, relevant sources were selected for detailed synthesis and inclusion. This strategy ensures the manuscript reflects foundational biology and the current (2026) landscape of dermatological nanomedicine.

## 2. Classification and Biogenesis of Bio-Nanovesicles

The therapeutic efficacy of BNVs is linked to their biogenesis, which determines size, surface marker expression, and cargo. Understanding these pathways is essential for translating laboratory findings into standardized clinical applications.

### 2.1. Natural Bio-Nanovesicles Subsets: Extracellular Vesicles (Exosomes and Microvesicles)

**Figure 1:**
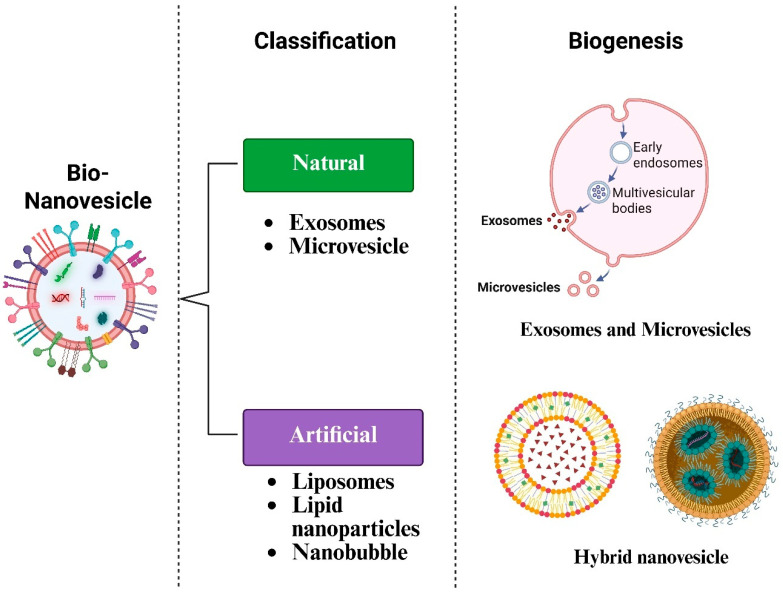
Classification and biogenesis of BNVs. BNVs are categorized as natural (exosomes and microvesicles) or artificial (liposomes, lipid nanoparticles, and nanobubbles). Exosomes originate from the endosomal pathway, whereas microvesicles bud directly from the plasma membrane. Hybrid nanovesicles combine biological membranes with synthetic lipids to enhance delivery. BNVs, bio-nanovesicles. Created in BioRender. Gangadaran, P. (2026) https://BioRender.com/yg8bo7m.

Natural BNVs: EVs (exosomes, microvesicles): Natural EVs are categorized based on biogenesis into exosomes and microvesicles. Exosomes (30–150 nm) originate from the endosomal system, where inward budding of MVB membranes forms intraluminal vesicles that are released into the extracellular space upon MVB-plasma membrane fusion [11]. Conversely, microvesicles (100–1000 nm) form through direct outward budding and pinching off of the plasma membrane (Figure 1). In skin and hair therapy, they serve as primary vehicles for paracrine signaling, allowing stem cells to communicate with target cells, such as the DP cells and keratinocytes, to initiate healing and growth cycles [19].

### 2.2. Engineered and Hybrid Nanovesicles: Overcoming Natural Limitations

Engineered/Artificial nanovesicles (e.g., EV mimetics, hybrid nanovesicles): To address limitations of natural EV production, such as low yield and inconsistent purity, researchers have developed engineered mimetics and hybrid systems. EV mimetics are typically produced through the mechanical disruption of donor cells (e.g., extrusion or microfluidics), resulting in nanovesicles that retain membrane proteins of the parent cells at higher concentrations than those of naturally secreted exosomes [20]. Hybrid nanovesicles combine biological and synthetic materials, often formed by fusing natural EVs with synthetic liposomes(Figure 1). This approach allows for a “best of both worlds” scenario: the biocompatibility and targeting capabilities of natural membranes integrated with the high drug-loading capacity and structural stability of synthetic nanoparticles [21,22].

### 2.3. Characterization as a Potency Assay

Key markers, isolation methods, and characterization: Accurate identification and purification of BNVs are essential for therapeutic consistency. Key markers include tetraspanins (CD63, CD81, and CD9) and endosome-associated proteins such as Alix and TSG101. Isolation methods vary in precision and scale, ranging from ultracentrifugation and size-exclusion chromatography to newer microfluidic and precipitation-based kits. Following isolation, BNVs were characterized using nanoparticle tracking analysis (NTA) to determine size and concentration, while transmission electron microscopy was used to confirm the characteristic cup-shaped morphology, ensuring vesicle integrity for clinical application [9,11,23].

Cargo composition (proteins, lipids, nucleic acids, and bioactive molecules): The therapeutic potency of BNVs is determined based on their diverse internal and membrane-bound cargo, which is selectively sorted during biogenesis. The protein fraction often includes signaling ligands and enzymes that directly modulate the microenvironment of the skin. The lipid bilayer is not merely a container; it contains bioactive lipids, such as sphingomyelin and cholesterol, that contribute to vesicle stability and cellular uptake. BNVs carry a rich “instructional manual” of nucleic acids, particularly miRNAs and long non-coding RNAs, which can epigenetically reprogram target skin cells to downregulate inflammation or upregulate hair follicle proliferation [24,25].

Having established the structural and biological foundations of BNVs, examining how these specific cargo profiles translate into regenerative signals within the hair follicle environment is critical.

## 3. Bio-Nanovesicles in Hair Regeneration: Mechanisms and Models

### 3.1. Cellular Targets: Dermal Papilla Cells, Hair Follicle Stem Cells

The efficacy of BNV-based therapy in hair regeneration depends on its ability to modulate two central cellular engines—DP cells and HFSCs. Located at the base of the hair bulb, DP cells act as the “command center” of the follicle, while BNVs, particularly those derived from mesenchymal stem cells (MSCs), deliver specific miRNAs (e.g., miR-218-5p) that activate the Wnt/β-catenin pathway in these cells. This activation maintains the inductive potential of DP cells, preventing follicle miniaturization and extending the anagen phase. Simultaneously, BNVs target HFSCs in the follicular bulge, delivering proteomic and genetic cues that activate these quiescent stem cells and trigger their proliferation and differentiation into the lineages required to form a new hair shaft [3,18,26]. Engineered fibroblast-derived nanovesicles can modulate DP cells to promote follicular regeneration and counteract alopecia in ex vivo human models [27,28].

Furthermore, BNV interactions with these targets establish a potent paracrine signaling loop that restores the microenvironment. Upon internalization by DP cells, BNVs stimulate immediate growth and induce secretion of endogenous growth factors, including Fibroblast Growth Factor-7 (FGF-7, keratinocyte growth factor) and vascular endothelial growth factor (VEGF). This secondary signaling promotes angiogenesis, increasing follicular blood flow, and reinforces hair germ structural integrity. By protecting HFSCs from apoptosis and enhancing DP inductive capacity, BNV-based therapies address the underlying cellular failures in conditions such as AGA, offering a dual-action mechanism that current evidence suggests is distinct from traditional chemical treatments [28,29,30].

### 3.2. Key Signaling Pathways

#### 3.2.1. Wnt/β-Catenin “Master Switch”

**Figure 2:**
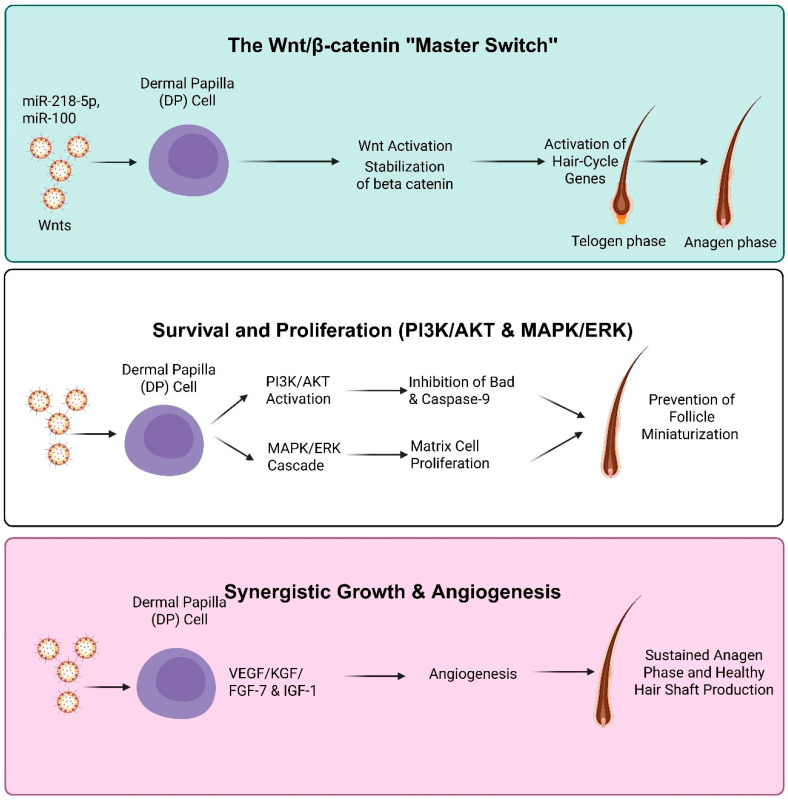
Mechanisms of BNV–mediated hair regeneration. BNVs activate the Wnt/β-catenin pathway in DP cells, promoting the telogen-to-anagen transition. They enhance PI3K/AKT and MAPK/ERK signaling, supporting cell survival and proliferation while preventing follicle miniaturization. Growth factors (VEGF, KGF, FGF-7, IGF-1) further stimulate angiogenesis, sustaining the anagen phase and healthy hair shaft growth. BNVs, bio-nanovesicles; DP, dermal papilla; Wnt, Wingless/Integrated; PI3K, phosphoinositide 3-kinase; AKT, protein kinase B; MAPK, mitogen-activated protein kinase; ERK, extracellular signal-regulated kinase; VEGF, vascular endothelial growth factor; KGF, keratinocyte growth factor; FGF-7, fibroblast growth factor-7; IGF-1, insulin-like growth factor-1. Created in BioRender. Gangadaran, P. (2026) https://BioRender.com/wbc9a2y.

Evidence indicates that new hair cycle initiation is associated with β-catenin stabilization. MSC-derived BNVs are often enriched in miR-218-5p and miR-100, which downregulate SFRP2 and DKK1 (endogenous Wnt inhibitors). By removing these inhibitory constraints, BNVs allow Wnt ligands to bind Frizzled receptors. This results in the inhibition of the GSK3β complex, preventing β-catenin degradation. Once stabilized, β-catenin translocates to the DP cell nucleus, activating hair-cycle-related genes and promoting the transition of the follicle from the telogen to the anagen phase [31,32,33]. Macrophage-derived EVs (MAC-EVs) enriched in Wnt3a and Wnt7b activate the Wnt/β-catenin pathway in DP cells, inducing Axin2 and Lef1 expression and promoting anagen initiation and hair follicle regeneration (Figure 2) [25].

##### Survival and Growth via PI3K/AKT and MAPK/ERK

Beyond initiating the hair cycle, BNVs may help sustain the follicle in the growth phase. By activating the PI3K/AKT pathway, BNV cargo induces phosphorylation of Bad and caspase-9, protecting DP cells from environmental stress and androgen-induced apoptosis. Simultaneously, activation of the MAPK/ERK signaling cascade stimulates the proliferation of hair matrix cells. This dual-action signaling enlarges the hair bulb, translating clinically into increased hair shaft diameter and reduced hair thinning (miniaturization) [25,26,34]. MAC-EVs enriched in Wnt3a and Wnt7b enhance DP cell proliferation and migration by activating PI3K/AKT and MAPK/ERK signaling pathways [25]. Additionally, fibroblast-derived engineered nanovesicles significantly upregulate PCNA, phosphorylated AKT, phosphorylated ERK, and vascular endothelial growth factor receptor 2 expression in DP cells (Figure 2) [26].

##### Transforming Growth Factor-Beta/Smad: Balancing Growth and Regression

The TGF-β superfamily acts as a double-edged regulator in the skin. While TGF-β2 induces the catagen phase and is implicated in AGA, BNVs can be engineered or naturally contain miRNAs (e.g., miR-122) that modulate this response. By inhibiting Smad2/3 phosphorylation, BNVs prevent premature follicular shutdown. Additionally, other members of this family, like BMP, are modulated by BNVs to maintain the quiescence of stem cells until the exact moment regeneration is required, ensuring the follicle does not exhaust its regenerative potential [17,35,36].

##### Synergistic Growth Factor Signaling

The physical growth of the hair fiber requires a substantial nutrient influx, facilitated by BNVs through the VEGF pathway. BNVs stimulate angiogenesis in surrounding endothelial cells, forming a dense capillary network around the follicle. Simultaneously, insulin-like growth Factor-1 (IGF-1) delivered or stimulated by BNVs acts as a potent keratinocyte mitogen, while FGF-7 functions as the primary paracrine signal from the DP to the hair matrix. Together, these pathways ensure that once the Wnt pathway is activated, the follicle has structural signals (FGF/IGF) and nutritional support (VEGF) to produce a healthy hair shaft [25,33]. MSC-EV/MAC-EV treatment increases IGF-1, VEGF, and KGF secretion, collectively triggering anagen entry and robust hair follicle growth(Figure 2) [25,33].

### 3.3. Preclinical Models

The transition of BNV-based therapies from laboratory to clinic requires robust preclinical models that simulate the complex architecture of the skin and hair follicle cycling [16,37]. These models enable the observation of BNV interactions with the stem cell niche in a multicellular environment.

#### 3.3.1. Human Hair Follicle Organ Culture

Ex vivo human hair follicle organ culture provides a critical bridge between cell culture and clinical trials. In this model, hair follicles are microdissected from scalp skin, often obtained from hair transplant surgeries, and maintained in a specialized medium. This allows direct assessment of BNV effects on hair shaft elongation rate and anagen-to-catagen transition, without confounding from systemic metabolism. Researchers use this model to track fluorescently labeled BNV penetration and to measure DP-derived growth factor secretion over time, providing high-fidelity data on human-specific responses to nanovesicle treatment [11,25,26,38].

#### 3.3.2. Mouse and Rat Models

Rodent models are the “gold standard” for studying the dynamic hair cycle and BNV safety in vivo. The telogen-to-anagen transition model, typically performed in C57BL/6 mice, is the most common assay. With synchronized hair cycles, these mice allow intradermal or topical BNV application to induce “precocious” anagen, characterized by the visible darkening of the skin from melanogenesis. Additionally, the wound-induced hair follicle neogenesis model is used to test the regenerative capacity of BNVs. This model shows whether BNVs can “reprogram” a healing wound to form new hair follicles rather than a scar, providing definitive evidence of their capacity to stimulate de novo appendage formation [32,33].

CNDs promote hair regeneration by delivering proangiogenic factors, miRNAs, and signaling molecules that stimulate DP activity, follicular proliferation, and induce anagen (Table 1).

While hair follicle cycling mechanisms are well-established, BNVs also affect inflammatory and chronic skin pathologies.

## 4. Bio-Nanovesicles in Dermatological Disease Therapy

BNV-based therapies have emerged as promising treatments for a range of dermatological conditions, surpassing traditional pharmacotherapy through multifaceted mechanisms such as immunomodulation, tissue regeneration, and targeted cellular crosstalk.

### 4.1. Inflammatory Skin Disease

EVs and exosomes from mesenchymal and other cells modulate immune responses in inflammatory skin conditions. MSC-derived exosomes reduce hyperinflammation in psoriasis models by downregulating Th17/Th1 cytokines, including TNF-α and IL-17 levels, thereby alleviating psoriatic lesions [45]. Similarly, adipose MSC exosomes (ASC-Exo) reduce psoriatic plaque severity and strengthen barrier function by suppressing pro-inflammatory signals [45]. In Th2-driven atopic dermatitis, stem cell exosomes suppress Th2, Th1, and Th17 cytokines. In a mouse model of atopic dermatitis, ASC-Exo injections significantly reduced Th2 cytokines (IL-4, IL-5, IL-13) and inflammatory mediators (TNF-α, IFN-γ, IL-17, and Thymic Stromal LymphoPoietin while restoring epidermal barrier ceramides and hydration [46]. These vesicles deliver bioactive RNAs and proteins associated with the reprogramming of keratinocytes and immune cells, shifting the cytokine milieu toward resolution. In vitro and preclinical studies identify BNVs as potent immunomodulators that normalize aberrant cytokine networks in psoriasis and atopic dermatitis, enabling cell-free therapeutic approaches [45,46,47].

### 4.2. Chronic Wounds and Burns

BNVs enhance wound repair through multiple mechanisms. EVs from stem or immune cells deliver pro-angiogenic factors (VEGF, FGF, EGF) and matrix-remodeling enzymes to injured tissue, accelerating neovascularization, granulation, and re-epithelialization [48]. For instance, macrophage-derived exosomes accelerate angiogenesis and re-epithelization while suppressing pro-inflammatory cytokines (TNF-α, IL-6) in diabetic wound models. Similarly, exosomes from fibroblasts or keratinocytes stimulate dermal cell proliferation and migration: fibroblast-derived EVs enhance VEGF and collagen production to form ECM and blood vessels, whereas keratinocyte-derived EVs trigger MAPK/ERK signaling to promote keratinocyte proliferation and wound closure. MSC-derived EVs reduce local inflammation and prevent chronic wound persistence by polarizing macrophages toward the reparative M2 phenotype and delivering anti-inflammatory miRNAs (e.g., miR-21, miR-146a). Together, enhanced angiogenesis, ECM remodeling, and re-epithelialization, and reduced injurious inflammation underlie the regenerative effects of EVs in various skin injuries, including burns. Engineered EV-mimetics and hybrid nanovesicles, enriched with specific growth factors, are being developed to enhance pro-healing activities, highlighting BNVs as versatile therapeutics for chronic wounds [48]. Fibroblast-derived EVs are promising cell-free therapeutics for wound healing and skin regeneration. Two studies evaluated EVs from primary human normal fibroblasts (hNF-EVs) and the L929 fibroblast cell line (L929-EVs).

Both EV types were well-characterized and enriched with proangiogenic cargo. hNF-EVs contained miR-130a, miR-210, VEGF-D, and CXCL8, while L929-EVs further promoted regenerative signaling. In vitro, fibroblast-derived EVs promoted fibroblast proliferation, migration, and wound-healing marker expression, and stimulated endothelial cell proliferation and tube formation. In mouse full-thickness wounds, scaffold-free or fibrin glue–assisted fibroblast-derived EV delivery accelerated wound closure, promoted collagen deposition and maturation, and enhanced angiogenesis (VEGF and CD31). Collectively, fibroblast-derived EVs promote angiogenesis and tissue remodeling, offering an effective cell-free strategy for skin wound healing [49,50,51]. MSC therapy holds promise for wound healing. 2D monolayer and 3D spheroid MSC cultures and their secretomes, including EVs in the conditioned media, were compared in this study. 3D-MSCs exhibited changes in survival and adhesion markers and secreted higher levels of IL-6, VEGFA, IL-8, and CXCL1. Their EV-rich conditioned medium promoted endothelial cell proliferation, migration, and tube formation, and markedly accelerated wound healing in a burn mouse model. Thus, 3D culture boosts the proangiogenic MSC secretome and EV content, enhancing therapeutic efficacy [52]. A comparative analysis of wound healing studies reveals that, while natural fibroblast-derived EVs promote collagen synthesis and angiogenesis, 3D MSC spheroid cultures produce a more potent secretome enriched with pro-angiogenic factors such as VEGFA and IL-8. Furthermore, the transition toward engineered EV-mimetics addresses the low-yield limitations of natural secretion, providing a scalable, standardized platform for delivering specific growth factors (VEGF, FGF) essential for rapid re-epithelialization in burn models.

### 4.3. Skin Aging and Pigmentation Disorders

EVs exert anti-aging and antioxidant effects by restoring youthful ECM and limiting oxidative damage in skin cells. In photoaging models, MSC-derived exosomes deliver growth factors and miRNAs that boost fibroblast collagen and elastin production while inhibiting matrix-degrading enzymes. For example, human umbilical cord blood MSC (hUCMSC)-derived exosomes enriched with EGF induced a time-dependent increase in dermal collagen I and elastin while downregulating Matrix Metalloproteinase-1. These vesicles stimulate endogenous antioxidant and anti-senescence signaling pathways; exosomal miR-291a-3p targets TGF-β receptor 2, protecting aged dermal fibroblasts from senescence and accelerating repair. By reducing reactive oxygen species and inflammatory cytokines in UV-stressed skin, BNVs help preserve skin elasticity and minimize wrinkles [53].

In pigmentation disorders, EV signaling between cells regulates melanocyte activity. Keratinocyte-derived exosomes enhance melanin synthesis by upregulating melanosomal enzyme expression and activity in melanocytes, amplifying pigmentation according to skin phototype and UV exposure. Paracrine EV transfer of regulatory miRNAs (e.g., miR-203) is believed to drive tanning and may contribute to hyperpigmentation disorders. Conversely, other BNV sources (e.g., stem-cell exosomes) may inhibit melanogenic cytokine signaling and melanin production, suggesting therapeutic potential for treating disorders such as melasma or vitiligo, though detailed data remain limited. Overall, EVs and engineered nanovesicles offer promising anti-aging and pigmentation-modulating strategies by delivering antioxidants, ECM-stabilizing signals, and melanocyte-targeting factors [54].

### 4.4. Scar Reduction and Fibrosis

In scarless or low-fibrosis healing, BNVs shift fibroblasts and myofibroblasts toward a regenerative phenotype. MSC-derived exosomes, especially from adipose tissue, downregulate TGF-β/Smad signaling in cutaneous fibroblasts, reducing myofibroblast markers and collagen deposition. For example, ADSC-derived exosomes inhibited the proliferation and migration of keloid fibroblasts while promoting apoptosis, accompanied by a dose-dependent decrease in α-SMA, TGF-β1, and Smad3 expression [55]. Similarly, exosome-treated wounds showed sparser collagen fibers and reduced fibrosis marker expression. At the matrix level, ADSC-derived exosomes promote scarless ECM remodeling by modulating collagen isoforms: they decrease fibroblast type-I collagen (Col-I) while increasing Col-III and MMPs (e.g., MMP3), normalizing the Col-III/Col-I ratio and enhancing matrix turnover [56]. These changes promote a pliable, well-structured dermal matrix over rigid scarring. In vivo, topical or injected exosomes accelerated wound closure and produced smaller and softer scars with reduced Col-I, Col-III, α-SMA, and phosphorylated Smad2/3 in the healed tissue. In sum, BNVs reduce hypertrophic scarring by inhibiting the fibrogenic TGF-β axis and restoring fibroblast activity and ECM production toward regeneration. In fibrosis, BNVs not only mask scar tissue but also reprogram the ECM toward a regenerative phenotype. ADSC-derived exosomes normalize the Col-III/Col-I ratio by inhibiting the fibrogenic TGF-β/Smad axis. This shift from rigid scarring to a pliable dermal matrix highlights the analytical advantage of BNVs over traditional topicals, providing the complex signals required to reset the tissue structure.

**Figure 3:**
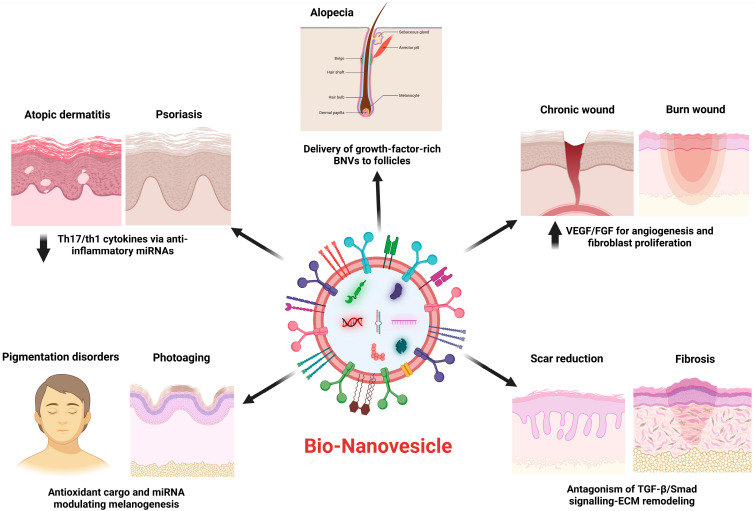
Illustration of BNVs in Dermatological Applications. BNVs deliver growth factors, miRNAs, and antioxidants to modulate inflammation (atopic dermatitis, psoriasis), promote hair regeneration in alopecia, enhance angiogenesis and fibroblast proliferation in chronic and burn wounds, regulate melanogenesis in pigmentation disorders and photoaging, and reduce fibrosis and scarring via TGF-β/Smad pathway modulation and ECM remodeling. BNV: Bio-nanovesicle; EVs: Extracellular vesicles; miRNAs: MicroRNAs; Th1: T helper 1; Th17: T helper 17; VEGF: Vascular endothelial growth factor; FGF: Fibroblast growth factor; TGF-β: Transforming growth factor-beta; ECM: Extracellular matrix. Created in BioRender. Gangadaran, P. (2026) https://BioRender.com/hlnfsvx.

BNVs promote hair regeneration, enhance wound healing, modulate inflammation, regulate pigmentation, and reduce fibrosis by delivering growth factors, miRNAs, and antioxidants (Figure 3).

## 5. Clinical Landscape and Translational Potential

### 5.1. Ongoing and Preclinical Trials

A growing number of early-phase clinical studies are evaluating exosome/EV-based therapies for dermatologic disorders, including hair loss. For AGA, several trials are ongoing or have recently been completed. For example, a Phase I/II trial (NCT05658094) investigating intradermal injections of human placenta-derived MSC exosomes in male and female patients with AGA (*n* ≈ 12) reports increases in hair density and shaft diameter, with no adverse events [42]. Another completed study (NCT06539273) at Yeditepe University (Turkey) evaluated intradermal injections of adipose- or foreskin-derived MSC exosomes in patients with AGA; however, the results have not yet been reported [57]. Ongoing trials include a U.S. Phase I study (NCT06482541; sponsor: Levit Dermatology) assessing Wharton’s jelly MSC-derived exosome solution in combination with scalp microneedling (planned *n* = 100) [57,58]. In addition, a Phase II trial in Pakistan (NCT06239207) is comparing intradermal injections of 2–10 × 10^9^ MSC-derived exosome particles (GFCCELL™ kit) with two sessions of autologous platelet-rich plasma in 30 patients with AGA. The primary endpoints include hair count and global physician and patient assessments at 6 months [58,59,60]. To date, no exosome-based therapies for alopecia areata have entered trials, though preclinical studies (e.g., hUCMSC-derived exosomes or baricitinib-loaded EVs in alopecia areata models) suggest accelerated regrowth and modulation of local inflammation [59,60].

Clinical trials of exosome-based therapies in skin diseases remain primarily early-phase. A Chinese Phase I trial (NCT05969717, Peking Union Medical College Hospital) is evaluating topical induced pluripotent stem cell (iPSC)-derived exosomes versus placebo in adults with moderate atopic dermatitis (Investigator Overall Assessment score 2–3, ≤5% body surface area) to assess safety and preliminary efficacy [61]. For chronic wounds, Exopharm Ltd. (Melbourne, Australia) conducted a first-in-human Phase I trial (ACTRN12620000944932) in which allogeneic platelet-derived EVs (LEAP-purified) were injected into induced acute wounds in healthy volunteers. The treatment was well-tolerated with no safety signals, although wound closure occurred in treated and control sites [62]. In psoriasis, a Phase I open-label trial (NCT05523011) is evaluating the safety of an MSC-derived exosome ointment (PTD-2021P) applied topically for 20 days in healthy volunteers [63]. Research on burn healing and pigmentation disorders remains largely preclinical or limited to pilot studies. Recent animal studies suggest that MSC-derived EVs incorporated into hydrogels or scaffolds enhance burn wound repair and reduce scarring by promoting fibroblast proliferation and collagen synthesis [64]. In a small clinical series (not an RCT), hUCMSC-derived exosomes combined with microneedling or fractional laser improved pigmentation scores in 60 patients with melasma versus laser + saline controls. Overall, human evidence remains limited and largely descriptive; however, animal-to-human translations are emerging, and ongoing trials may help clarify the feasibility of exosome-based therapies in these conditions [65]. Multiple early-phase clinical studies have evaluated extracellular vesicle (EV)-based therapies across dermatological indications (Table 2)

### 5.2. Barriers to Clinical Translation

#### 5.2.1. Methodological Heterogeneity and the “Purity vs. Yield” Trade-Off

A major limitation in the current literature is the lack of standardized isolation protocols, which complicates the reproducibility of BNV-mediated effects. Although ultracentrifugation remains a commonly used “gold standard,” it can promote vesicle aggregation and co-isolation of non-vesicular protein contaminants, potentially confounding mechanistic studies [66]. In contrast, size-exclusion chromatography provides higher purity but often at the cost of lower yield. Consequently, studies using ostensibly the same MSC-derived exosomes may report conflicting results simply due to differences in the purity of the isolates.

#### 5.2.2. Standardization Crisis in Characterization

Currently, a universal consensus on what constitutes a “standard dose” of BNVs is unavailable. Most studies rely on NTA for particle quantification; however, this method cannot distinguish functional exosomes from similarly sized protein aggregates or “ghost” vesicles. Without standardized potency assays that assess biological activity rather than particle presence, clinical translation remains limited. International organizations such as the International Society for Extracellular Vesicles recommend using specific marker panels (e.g., CD63, CD81, Alix), but adherence across the literature remains inconsistent, contributing to a body of evidence that is more descriptive than reproducible [51,67].

#### 5.2.3. Regulatory and Clinical Reality Considerations

Despite considerable interest in BNVs in aesthetic medicine, no FDA-approved exosome-based products for hair or skin regeneration are currently available. Many products marketed as “exosomes” in clinical settings are categorized as cosmeceuticals, bypassing the rigorous safety and efficacy testing required for biological therapies. The risk of off-target effects and uncertain long-term biodistribution, particularly hepatic or splenic accumulation, remains an important challenge for systemic applications [68].

### 5.3. Comparative Advantages over Stem Cells and Traditional Biologics

BNVs combine features of cell therapies and molecular biologics while potentially mitigating certain risks.

Safety/Immunogenicity: Unlike live stem cells (e.g., MSCs or iPSCs), EVs cannot divide or engraft, thereby reducing concerns about ectopic tissue formation or malignant transformation. Consistent with this distinction, both clinical and preclinical studies consistently report minimal toxicity and no tumor formation following exosome treatment [42,62]. MSC-derived EVs lack major histocompatibility complex class II expression and have low immunogenicity [69], potentially permitting allogeneic “off-the-shelf” use without immunosuppression, in contrast to cell grafts.

Delivery and Targeting: EVs naturally carry diverse bioactive molecules (e.g., proteins and miRNAs) and are readily internalized by recipient cells, facilitating intracellular delivery that free growth factors cannot achieve. For instance, platelet-derived EVs contain IGF and TGF-β that are associated with angiogenesis and fibroblast proliferation [62]. EVs can also traverse tissue barriers; in the skin, penetration may be enhanced by micro-injury or delivery carriers. They are also more resistant to proteolytic degradation than free cytokines, extending bioactivity. Engineered exosomes can also be modified with targeting ligands to promote cell-specific delivery, providing a modular advantage over recombinant proteins.

Manufacturing and Scalability: Although EV production is complex, it may scale more readily than cell-based therapies. Producer cells can be expanded in bioreactors to continuously release EVs, which can then be concentrated and stored without maintaining viability. This contrasts with cell therapies requiring cryopreservation of viable cells. Once a good manufacturing practice-compliant process is established, batch production cost may be lower than that of autologous or pluripotent cell products. Simultaneously, EV-based approaches avoid ethical concerns associated with embryonic stem cells and the genetic modification issues linked to iPSCs.

Other Advantages: Similar to biologics (e.g., recombinant growth factors), EVs can be manufactured as defined therapeutic products while delivering complex molecular cargo and signaling networks in one platform. In this sense, EVs may preserve the therapeutic properties of their parent MSCs while avoiding risks associated with live-cell therapies. Comparative analysis in the literature also suggests that EV-based therapies produce durable anti-inflammatory effects similar to MSCs but with simpler storage and transport requirements [66]. Together with their low oncogenic potential, these features suggest a favorable safety and cost profile relative to stem-cell transplantation and high-dose protein biologics.

The selection of a BNV source is a strategic decision determined by the target pathology rather than simple availability. For instance, MSC-derived BNVs are often the “gold standard” for inflammatory conditions such as psoriasis because of their cytokine-modulating cargo, whereas engineered mimetics are increasingly explored for alopecia treatments where high-dose, standardized delivery across the stratum corneum is required. The previously noted standardization challenge is further complicated by evidence that the same cell source can produce different vesicle profiles depending on culture conditions, such as 2D monolayers or 3D spheroids, with the latter often yielding vesicles with greater pro-angiogenic activity. Therefore, more systematic source selection—potentially supported by artificial intelligence (AI)-driven cargo profiling—may be required to achieve reproducible clinical outcomes. BNV sources exhibit distinct advantages and limitations that influence their translational potential (Table 3)

### 5.4. Industry Collaborations and Biotech Interest

The BNV field has attracted notable investment and industry partnerships, especially in regenerative medicine and aesthetic applications. Companies such as Codiak Biosciences (USA) and Evox Therapeutics (UK) have raised significant venture funding to support exosome-based drug development (Evox ~$95 million in 2021 [70]) and established pharmaceutical collaborations (e.g., Sarepta–Codiak for CNS delivery, Takeda/Eli Lilly–Evox for CNS and rare diseases). Although these programs are not dermatology-focused, they demonstrate broader industry confidence in EV platforms. In cosmetic dermatology, numerous companies now market exosome-containing products. For example, Benev (USA), Lifeline, SkinMedica, and others market MSC-derived exosome serums or creams for skin rejuvenation [25,71]. Clinics routinely offer exosome treatments, often combined with microneedling or lasers, for anti-aging, scarring, and hair loss. A recent U.S. product launch by ExoCel Bio introduced a placental MSC-derived exosome hair serum (“Refine”, 75 billion vesicles) for androgenic alopecia [72]. Similarly, RION Aesthetics markets platelet-derived EV hair serums with adjuvants. Market analyses suggest strong commercial interest. For example, BioInformant estimated the global exosome skincare market at ~$250 million and growing [73]. Concurrently, investment continues through direct-to-consumer cosmeceutical ventures, technology licensing (e.g., EV platforms developed by Exopharm Ltd. Melbourne, Australia), and partnerships in regenerative medicine. Together, these collaborations—from biotechnology research to commercial aesthetics—underscore substantial industry interest in translating BNV biology into dermatologic and regenerative products.

## 6. Conclusions and Future Perspectives

Recent advances in regenerative medicine highlight the therapeutic potential of cell-derived EVs, including exosomes and exosome-mimetic nanovesicles, for hair follicle regeneration and inflammatory skin conditions [3]. EVs, particularly those from MSCs or neural progenitor lines, show enhanced DP cell proliferation and stimulate hair follicle cycling. This activity is partly attributed to modulation of the Wnt/β-catenin signaling pathway, with enrichment of miR-100 identified as a key contributor [3,71]. In experimental models, nanovesicles produced via cell extrusion—such as those derived from human neural progenitor cell lines—accelerated entry into the anagen phase and improved follicle density and shaft thickness [71].

Combinatorial nanovesicle formulations have further broadened therapeutic potential. For example, resveratrol-loaded lipid nanovesicles (PPD-Lip@RES) combine antioxidant, anti-inflammatory, and pro-angiogenic effects. In vivo studies report enhanced hair coverage, increased shaft thickness, and upregulated regenerative markers, including Wnt3a, β-catenin, and VEGF, alongside suppression of inhibitory pathways such as DKK-1 and TGF-β1 [74]. These findings suggest a multifaceted mechanism supporting follicle restoration and microenvironmental homeostasis.

Parallel progress has been made in skin disease therapy, where phospholipid-based soft vesicles—including ethosomes, transfersomes, and niosomes—have emerged as effective dermal drug carriers. These systems enhance skin penetration and controlled release, benefitting chronic conditions such as psoriasis and acne [75]. Plant-derived nanovesicles (PDNVs), especially when incorporated into hydrogels, demonstrate anti-inflammatory effects in preclinical models [76]. Recent approaches combining PDNVs with metal oxide nanoparticles (e.g., ZnO, CeO_2_, Ag) and botanical extracts in topical gels show accelerated wound healing and reduced inflammation, highlighting a promising synergy between nanocarriers and phytochemicals [77].

Integration of these nanotechnologies with emerging platforms such as 3D bioprinting and organoid systems represents a substantial advance. Nanovesicles embedded within bioprintable bioinks or organoid cultures can guide stem cell differentiation, enhance vascularization, and support functional tissue regeneration [78]. These engineered constructs are increasingly applied in disease modeling and therapeutic testing, enabling more predictive and physiologically relevant platforms [79].

AI further enhances these capabilities by enabling data-driven design, optimization, and analysis. In bioprinting, AI-guided frameworks improve parameter tuning and process reproducibility [80]. AI-enabled organoid platforms derived from patient-specific cells allow simulation of disease states and prediction of individual responses to nanovesicle-based therapies [80]. Moreover, AI can guide rational nanovesicle design, refining characteristics such as size, charge, and cargo composition to improve targeting and biodistribution. Future perspectives point toward highly personalized nanotherapeutic strategies. Integration of AI with multi-omics profiling from patient-derived organoids may enable highly personalized nanotherapeutic strategies tailored to individual gene expression or miRNA profiles.

Unlike previous reviews that focused primarily on the paracrine effects of natural MSC-exosomes, this perspective emphasizes synthetic-biological hybrids and AI-optimized delivery systems. Incorporating BNVs into 3D-bioprinted scaffolds and using patient-derived organoids for predictive modeling represent a new conceptual advance in dermatology. These technologies collectively offer a transformative, patient-specific approach to regenerating skin and hair structures.

## Figures and Tables

**Table 1:** Cell-derived nanovesicles for hair regeneration therapy.

BNV Source	Study Type & Model	Delivery Route	Key Bioactive Cargo	Primary Mechanism	Therapeutic Outcome	References
DPCs	In vitro (Human DPCs)	Media supplement	miR-218-5p, miR-195-5p	Activation of Wnt/β-catenin signaling; upregulation of pAKT and downregulation SFRP2.	Restores hair-inductive potential; reverses follicle miniaturization in AGA.	[18]
Bone Marrow-MSCs	In vivo (C57BL/6 mice)	Intradermal injection	Unknown	Increased expression and secretion of VEGF and IGF-1; Activation of PI3K/AKT and MAPK/ERK cascades.	Promotes hair follicle conversion from telogen to anagen in mice	[33]
ADSCs	In vivo (Mouse model)	Topical/Injection	VEGF, PDGF, miR-122	Promotion of angiogenesis and modulation of the TGF-β pathway.	Increases hair density and thickness; comparable efficacy to 5% Minoxidil.	[39]
Macrophage	In vivo (Mice) & Ex vivo (Human HF)	Injection/Organ culture	Wnt3a and Wnt7b	Activation of Wnt/β-catenin/AKT, increased expression of VEGF & KGF	Increases hair density and thickness in mice and improves hair elongation in human HF.	[25]
FB	Ex vivo (Human HF organ culture)	Media supplement	Wnt3a	Activation of Wnt/β-catenin; elevating expression of the target genes Axin2 and Lef1	promote hair growth in cultured human hair follicles	[11]
hUC-MSCs	In vivo (C57BL/6 mice)	Intradermal injection	miR-146a-5p, Wnt ligands	Upregulation of RAS/ERK and inhibition of SFRP1.	Accelerates telogen-to-anagen transition; promotes DP cell migration.	[40]
FBs	Ex vivo (Human hair follicle organ culture)	Culture media supplement	VEGF-A, p-AKT	Activation of PI3K/AKT and MAPK/ERK cascades.	Enhances DP cell proliferation; increases human hair shaft length ex vivo.	[41]
Placenta-Derived MSCs	Clinical trial (Phase I/II, n≈12)	Intradermal injection	Growth factors, Cytokines	Immune privilege restoration; suppression of apoptosis in follicular cells.	Significant clinical increase in hair density (e.g., from 96 to 163 hairs/cm^2^).	[42]
Plant-Derived (e.g., Ginseng, Ginger)	In vivo (Mouse wound-induced neogenesis)	Topical gel	Plant-specific miRNAs, lipids	Scavenging of ROS; anti-inflammatory effects.	Stimulates hair follicle neogenesis; reduces oxidative stress in the scalp.	[43]
Engineered Mimetics (EV Mimetics)	In vivo (Murine models)	Targeted transdermal	Loaded Finasteride, miR-214	Targeted transdermal delivery; sustained release of high-potency cargo.	Superior skin permeation; robust stimulation of hair regrowth in murine models.	[44]

BNV: Bio-Nanovesicle; DPCs: Dermal Papilla Cells; miR: microRNA; Wnt: Wingless/Integrated signaling pathway; β-catenin: Beta-catenin; vcan: Versican gene; AGA: Androgenetic Alopecia; MSCs: Mesenchymal Stem Cells; VEGF: Vascular Endothelial Growth Factor; IGF-1: Insulin-Like Growth Factor-1; PI3K: Phosphoinositide 3-Kinase; AKT: Protein Kinase B; MAPK: Mitogen-Activated Protein Kinase; ERK: Extracellular Signal-Regulated Kinase; ADSCs: Adipose-Derived Stem Cells; PDGF: Platelet-Derived Growth Factor; TGF-β: Transforming Growth Factor-Beta; KGF: Keratinocyte Growth Factor; HF: Hair Follicle; Axin2: Axis Inhibition Protein 2; Lef1: Lymphoid Enhancer-Binding Factor 1; hUC-MSCs: Human Umbilical Cord-Derived Mesenchymal Stem Cells; RAS: Rat Sarcoma viral oncogene; SFRP1: Secreted Frizzled-Related Protein 1; FBs: Fibroblasts; VEGF-A: Vascular Endothelial Growth Factor A; p-AKT: Phosphorylated AKT; ROS: Reactive Oxygen Species; EV: Extracellular Vesicle.

**Table 2:** Summary of studies on cell-derived nanovesicles for skin disease therapy.

Skin Condition	Study ID	Vesicle Source	Delivery Method	Design and Participants	Phase	Key Outcomes
AGA	NCT05658094	Human placenta derived MSC exosomes	Intradermal injection	n ≈ 12 male & female patients	Phase I/II	Increased hair density & diameter; no adverse events
AGA	NCT06539273	Adipose- or foreskin-derived MSC exosomes	Intradermal injection	Patient number not specified	Phase I/II	Results pending
AGA	NCT06482541	Wharton’s jelly MSC exosome solution	Microneedling + topical exosome application	Planned (n = 100)	Phase I	–
AGA	NCT06239207	MSC exosomes (2–10 × 10^9^ particles)	Intradermal injection	30 AGA patients; vs. 2 sessions autologous PRP	Phase II	Primary: hair count & global physician/patient assessment at 6 mo
AD	NCT05969717	iPSC-derived exosomes (GD-iExo-001)	Topical application	Moderate AD adults (IGA 2–3; ≤5% BSA); vs. placebo	Phase I	Safety & preliminary efficacy assessed
Chronic wounds	ACTRN12620000944932	Allogeneic platelet-derived EVs	Local injection into acute wounds	Healthy volunteers with induced wounds	Phase I	Well-tolerated; wound closure equivalent in treated and control sites
Psoriasis	NCT05523011	MSC exosome ointment (PTD-2021P)	Topical ointment for 20 days	Healthy volunteers	Phase I (open-label)	Safety evaluated; data pending
Melasma (pigmentation)	Clinical series	hUCMSC exosomes	Microneedling or fractional laser + exosomes	60 patients; non-randomized	Pilot clinical series	Improved pigmentation scores vs. laser + saline controls

AGA, Androgenetic Alopecia; MSC, Mesenchymal Stem Cell; iPSC, Induced Pluripotent Stem Cell; EVs, Extracellular Vesicles; PRP, Platelet-Rich Plasma; AD, Atopic Dermatitis; IGA, Investigator’s Global Assessment; BSA, Body Surface Area; hUCMSC, Human Umbilical Cord Mesenchymal Stem Cell; NCT, National Clinical Trial Identifier (ClinicalTrials.gov); ACTRN, Australian New Zealand Clinical Trials Registry Number.

**Table 3:** Bio-nanovesicle sources: comparative advantages and limitations.

BNV Source	Primary Advantages	Technical & Clinical Limitations
MSC-derived (bone marrow/adipose)	High regenerative potency; well-characterized immunomodulatory cargo (miRNAs). Low immunogenicity (MHC class II negative).	Significant donor-to-donor variability. Scaling is limited by the finite expansion capacity of primary cells.
Fibroblast-derived	Superior for collagen synthesis and matrix remodeling. High compatibility for “self-to-self” autologous skin applications.	Lower anti-inflammatory potency compared to MSCs. Slower proliferation rates in 2D culture.
Plant-derived (exosome-like)	Highly scalable and cost-effective. Rich in antioxidant phytochemicals and “natural” lipids.	Potential cross-species immune reactions. Cargo varies significantly based on environmental growth conditions.
Engineered mimetics (CDNs)	Extremely high yield (up to 100-fold higher than natural secretion). Modular: can be custom-loaded with drugs (e.g., Finasteride).	Fragmentation process may alter membrane protein orientation. Requires specialized microfluidic or extrusion equipment.
iPSC-derived	Virtually unlimited supply due to cell immortality. High consistency and batch-to-batch reproducibility.	High manufacturing costs and stringent regulatory oversight. Lingering (though low) concerns regarding pluripotency markers.

BNV, bio-nanovesicles; MSC, mesenchymal stem cell; MHC, major histocompatibility complex; 2D, two-dimensional; CDN, cell-derived nanovesicles; iPSC, induced pluripotent stem cell.

## Data Availability

No new datasets were generated or analyzed in this study. All data discussed are derived from previously published articles cited in the reference list.
